# Colorectal cancer in a man with silver-Russell syndrome: a case report

**DOI:** 10.1093/omcr/omaf204

**Published:** 2025-10-29

**Authors:** Farah Ibraik, Ibraheem Hammouri, Hasan Arafat, Ahmad G Hammouri, Hanna Qahoush

**Affiliations:** Faculty of Medicine and Health Sciences, Al-Najah National University, Nablus, West Bank, Palestine; Clinical Research & Teaching Assistant, Faculty of Medicine and Health Sciences, Palestine Polytechnic University, Hebron, West Bank, Palestine; Department of Internal Medicine, Augusta Victoria Hospital, Jerusalem, Palestine; Radiology Department, Al-Ahli hospital, Hebron, West Bank, Palestine; Department of Internal Medicine, Augusta Victoria Hospital, Jerusalem, Palestine

**Keywords:** silver-Russell syndrome, Russell-silver syndrome, colorectal cancer, imprinting disorder, case-report

## Abstract

Silver-Russell syndrome (SRS), also known as Russell-Silver syndrome (RSS), is a congenital growth disorder characterized by intrauterine and postnatal growth retardation, craniofacial disproportion, asymmetry, and other distinctive features. It was first described by Alex Russell and Henry Silver in the 50s of the 20th century. Its broad range of manifestations makes its true incidence difficult to determine. While gastrointestinal anomalies such as gastroesophageal reflux disease and esophagitis have been reported in SRS patients, an association with colorectal cancer (CRC) has not been described. Here, we present the case of a 31-year-old man with SRS who was referred to us for constipation. A rectal biopsy revealed moderately differentiated adenocarcinoma, and a staging CT scan demonstrated multiple lung and hepatic nodules.

## Introduction

Silver-Russell syndrome (SRS) is a rare imprinting disorder characterized by intrauterine growth retardation, restricted postnatal growth, relative macrocephaly, body asymmetry, and distinctive facial dysmorphism [1, 2]. Both clinical and genetic characteristics can vary among patients. The mode of inheritance is variable, and most cases are sporadic. Since its description in 1954, around 400 cases of SRS have been reported [3].

To the best of our knowledge, this is the first case to be reported of colorectal adenocarcinoma in a patient with SRS.

## Case presentation

A 31-year-old man presented with complaints of constipation and fresh blood per rectum of two months duration. The patient had been previously diagnosed with SRS by genetic testing in early childhood by a pediatric specialist, according to family report. While the original genetic testing records were unavailable due to limited medical record preservation in his rural area, the patient’s phenotype was consistent with the Netchine–Harbison clinical scoring system (NH-CSS) for Silver–Russell syndrome [4], with features including history of intrauterine growth restriction, postnatal growth failure, relative macrocephaly, triangular face, and limb length asymmetry. These features persisted into adulthood and were evident during our assessment. The patient has a brother diagnosed with SRS as well.

His medical history included poor vision, hearing impairment and learning difficulties, which led to his early dropout of school. The patient had no known risk factors for colorectal cancer, apart from active smoking.

On physical examination, he had ascites. His height and weight were 154 cm and 25 kg, respectively. His body mass index (BMI) of 10.54 indicated that he was underweight. Facial dysmorphism, characterized by a triangular face, hypertelorism, widely-spaced teeth, low-set ears, scanty eyebrows, a depressed nasal bridge, and frontal bossing, was also noted.

Basic investigations included a complete blood count (CBC), kidney function tests, glycated hemoglobin (HbA1c), and thyroid function tests that were within normal limits. Carcinoembryonic antigen (CEA) was positive with a value of 12.08 ng/mL. However, his CA 19–9 was negative.

**Figure 1:**
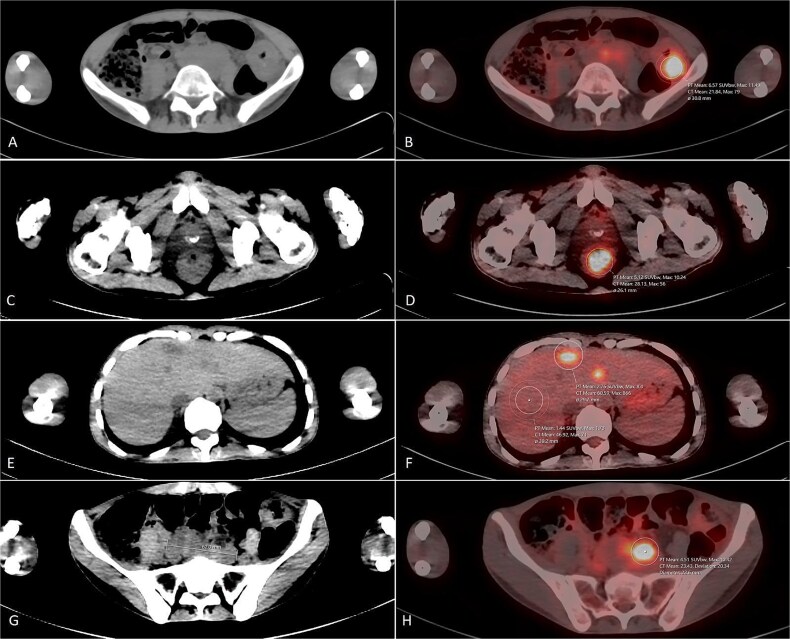
F-18 FDG PET/CT scan for the patient. Selected CT axial cuts with the corresponding PET/CT fusion images. An evidence of intensely hyper-metabolic left colonic mass is noted (A & B), in addition to another concentric intensely hyper-metabolic rectal mass just above the anal verge (C & D). Few hypo-attenuating lesions with high grade uptake were seen in the liver (E & F) as well as multiple enlarged intensely hyper-metabolic retro-peritoneal lymph nodes (G & H).

Echocardiography revealed normal results with an ejection fraction (EF) of 65%. Colonoscopy revealed two masses (one was 1 cm away from the anal verge, and the other was located at the hepatic flexure). A rectal biopsy revealed a moderately differentiated adenocarcinoma. Staging CT scan revealed multiple colon polyps, few bilateral lung nodules and multiple hepatic nodules.[Fig. 1.] The tumor was negative for the common BRAF, KRAS, and NRAS mutations.

Following diagnosis of rectal adenocarcinoma, the patient was initiated on CAPEOX chemotherapy. The first cycle was administered with a dose reduction, and the patient was scheduled for reassessment after three weeks to evaluate treatment tolerance and consider dose escalation. However, the patient was subsequently lost to follow-up and did not return to the hospital for subsequent care.

## Discussion

Silver-Russell syndrome (SRS) is a rare imprinting disorder characterized by intrauterine growth retardation, restricted postnatal growth, relative macrocephaly, body asymmetry, and specific facial dysmorphism [1, 2]. These include a triangular facial appearance, a prominent forehead, and a downward curvature of the mouth [5].

Since its description in 1954, around 400 cases of SRS have been reported, with a prevalence of 1/30000–1/100000 [3, 6]. SRS is probably more common than has been suggested, but its broad range of clinical features makes it easy to miss and harder to diagnose.

SRS is both genetically and clinically heterogeneous. The mode of inheritance is variable, and most cases are sporadic. However, SRS-associated molecular anomalies have been studied, and maternal uniparental disomy of chromosome 7 is observed in approximately 10% of patients [3]. Around 50% have loss of methylation (LOM) of the telomeric imprinting control region (H19/IGF2:IG-DMR or imprinting center 1 (IC1)) on 11p15.5 [7].

Although SRS isn’t a cancer predisposition syndrome by itself—on the contrary to other rare imprinting disorders such as Beckwith-Weidemann syndrome, for example [8]—some tumors have been reported in SRS patients over the years, including testicular cancer, hepatocellular carcinoma, craniopharyngioma, and supratentorial juvenile pilocytic astrocytoma [9].

To the best of our knowledge, this is the first case of colorectal cancer—let alone an early-onset one—in a SRS patient to be reported in the English literature. No association has been previously made between SRS and colorectal cancer. However, a recent study conducted on 25 SRS patients with a median age of 32 years concluded that these patients had an unfavorable body composition and predisposition to cardiometabolic disease based on their body fat percentage and regardless of their BMI [10]. This result supports that of another smaller study conducted on 7 adult patients, two of whom turned out to have glucose intolerance and hyperinsulinemia, and two showed a high total cholesterol level with low high-density lipoprotein (HDL) cholesterol levels [11]. These frequently overlooked metabolic abnormalities can be risk factors for developing colorectal cancer in such patients and at such a young age.

Our second hypothesis is on the molecular level and involves the common and specific 11p15 Imprinting Center Region 1 Loss of Methylation. This region controls the expression of the insulin-like growth factor 2 (IGF2) gene [12]. In the physiologic setting, IGF2 works as a growth-promoting hormone during gestation. However, as a mitogen, it is associated with an increased risk of developing colorectal neoplasia. Of course, colorectal cancer is a highly heterogeneous tumor, and it’s hard to elucidate its progression and the mutations accounting for it [13]. Nonetheless, we think this possibility is worth further investigation.

Most cases of early-onset colorectal cancer (EOCRC) are associated with hereditary cancer syndromes such as familial adenomatous polyposis or other germline mutations [14]. In our patient, the tumor was negative for the common BRAF, KRAS, and NRAS mutations. Additionally, the patient had no history of inflammatory bowel disease or other conditions known to predispose to colorectal cancer, and there was no family history of colorectal or related cancers. The only identifiable risk factor was active smoking, suggesting a potentially distinct association with SRS rather than typical EOCRC.

Currently, no specific CRC screening guidelines exist for individuals with SRS, either internationally [15] or within the Palestinian Ministry of Health. This case highlights the need for further research into colorectal cancer risks in SRS patients.

## Conclusion

We report here a rare case of colorectal cancer in a young man with Silver-Russell syndrome. To our knowledge, this is the first report of colorectal cancer with SRS. Further research and molecular studies are required to confirm the association between SRS and colorectal cancer before definitive recommendations can be made.
